# Changes of immune-related factors in the blood of schizophrenia and bipolar disorder patients receiving monotherapy

**DOI:** 10.1038/s41398-022-01968-0

**Published:** 2022-05-26

**Authors:** Fangyuan Duan, Shunan Zhao, Cuihua Xia, Zongyao Ren, Ning Yuan, Li Xie, Le Wang, Yifan Xiong, Pei Yu, Yu Chen, Jianghua Tian, Jiacheng Dai, Jiaqi Lu, Yan Xia, Xuejun Liu, Chao Chen, Chunyu Liu

**Affiliations:** 1 Center for Medical Genetics & Hunan Key Laboratory of Medical Genetics, School of Life Sciences, and Department of Psychiatry, The Second Xiangya Hospital, Central South University, Changsha, China; 2 School of Psychology, Shaanxi Normal University, Xi’an, Shaanxi China; 3 Department of Psychiatry, Hunan Provincial Brain Hospital, Clinical Medical School of Hunan University of Chinese Medicine, Changsha, Hunan China; 4 Department of Psychiatry, SUNY Upstate Medical University, Syracuse, NY USA; 5 National Clinical Research Center on Mental Disorders, The Second Xiangya Hospital, Central South University, Changsha, China; 6 Hunan Key Laboratory of Animal Models for Human Diseases, Central South University, Changsha, China

**Keywords:** Molecular neuroscience, Predictive markers

## Abstract

Schizophrenia (SCZ) and bipolar disorder (BPD) are associated with abnormal expression of immune-related factors (IRFs), which have been proposed as biomarkers of either disease diagnosis (trait markers) or treatment (state markers). However, the state markers have been found to be less reproducible than the trait markers in previous studies. In the current study, we focused on the changes of IRFs in blood of SCZ and BPD patients receiving monotherapy. SCZ (*N* = 49) and BPD (*N* = 49) Chinese patients were recruited at acute episode and followed for 9 to 51 days until remission. Blood samples were collected at two state-points, acute state before treatment and remission state after treatment. A total of 41 IRFs in plasma were quantified by the Luminex assay. After adjusting covariates, we found four cytokines or cytokine receptors were significantly increased at remission when compared to acute episode in all the patients, including CD30, BAFF, CCL20, and CXCL10 (Bonferroni corrected *p* < 0.05). CD30 and BAFF were consistently increased in both SCZ and BPD while the increase of CCL20 was only observed in BPD but not SCZ when analyzing the two disorders separately. CXCL10 change was not significant in either SCZ or BPD alone. The changes of these four factors were correlated with each other, but not with clinical features. CD30 concentration in the BPD acute state was correlated with sleep quality (Spearman’s *r*_*s*_ = 0.365, Bonferroni corrected *p* < 0.05). Overall, we found that four factors (CD30, BAFF, CCL20, and CXCL10) might be associated with treatment of psychosis.

## Introduction

Schizophrenia (SCZ) and bipolar disorder (BPD) are psychiatric disorders characterized by psychotic symptoms, cognitive impairment, and functional decline [1, 2]. Both disorders cause severe disability, generate high social and economic burden, and have high prevalence rate [1–4]. These two disorders share genetic risk loci [5, 6], clinical features, and treatments [7–9]. Immune system dysregulation has been implicated in the development and progression of SCZ and BPD [10, 11]. Change in immune-related factors (IRFs) in these patients is an important feature of these disorders [12, 13].

Biomarkers are necessary to prevent, treat, and monitor disorders. Trait markers and state markers are two major classes of biomarkers for psychiatric disorders such as SCZ and BPD [14]. Trait markers are stable during disease progression and treatment, reflecting predispositions to the diseases. For example, SCZ and BPD patients had a significantly increased level of tumor necrosis factor (TNF)-α in blood compared with healthy controls and this factor was stable during treatment [15]. In contrast, state markers are dynamic during disease progression and may reflect symptomatic changes. For example, a meta-analysis of 40 studies of SCZ showed that the interleukin (IL)-1β, IL-6, and tumor growth factor (TGF)-β levels in blood and cerebrospinal fluid (CSF) were increased in the acute state and normalized in remission after treatment [16]. In BPD, another meta-analysis investigated the changes of brain-derived neurotrophic factor (BDNF) during different mood states and concluded that BDNF level in serum and plasma was increased after treatment for acute mania [17].

Despite numerous reported alterations of IRFs in SCZ and BPD, it is worth noting that the state markers were less reproducible than trait markers according to our review [18]. One of the major causes of poor reproducibility is related to underpowered sample size. There are far fewer well-powered studies of state markers than the case-control studies of trait markers. Most previous studies of state markers were based on cross-sectional data, comparing patients at different clinical states with controls, rather than a longitudinal design comparing the same subjects before and after treatment. Cross-sectional design is highly vulnerable to individual heterogeneity, such as disease pathology, body mass index, smoking, age, sex, and metabolic syndromes [19], which could reduce statistical power.

In the present study, we aim to use longitudinal design to identify the state markers from a multiplex immunoassay panel of 41 IRFs in a cohort of SCZ and BPD. The SCZ and BPD patients were enrolled in acute episode and were followed for 9 to 51 days until they went into remission after monotherapy. We measured abundance of IRFs in these patients before and after treatment.

## Methods

### Participants

All subjects were recruited from Hunan Provincial Brain Hospital between July and November 2017. Inclusion criteria for SCZ and BPD were: (1) diagnosis of SCZ or BPD based on the Diagnostic and Statistical Manual of Mental Disorders-Fifth Edition (DSM-5) criteria and (2) age between 16 and 60 years. Diagnosis of the patients was made by two psychiatrists. Study exclusion criteria were: (1) major psychiatric illness history other than SCZ or BPD; (2) severe physical illness (e.g., cardiovascular and cerebrovascular, liver, kidney, blood, or endocrine diseases) and history of autoimmune diseases; (3) alcohol or drug abuse; (4) vaccination or medication that could affect immune function within one month, acute infection (for example, cold and fever) within the past two weeks; (5) pregnancy, breastfeeding, and menstrual periods for females at sampling point.

The study enrolled 49 SCZ and 49 BPD patients who were all in acute episode before treatment. Of the BPD patients, 40 were diagnosed with mania and 9 with depression. During the follow up, 7 patients with SCZ and 9 patients of BPD dropped out. During the treatment, one BPD patient experienced mania, depression, and euthymia. Tables 1 and 2 outline demographic characteristics and clinical status for the patients. These patients received monotherapy with either antidepressant, mood-stabilizer, antipsychotic medications or other drugs according to their clinical situation. The most frequently used drugs for SCZ were magnesium valproate (18 patients, 42.9%), risperidone (18, 42.9%) and olanzapine (16, 38.1%). The most frequently used drugs for BPD were lithium (22, 55.0%), magnesium valproate (21, 52.5%) and quetiapine fumarate (21, 52.5%). SCZ and BPD participants were followed up for 9~51 days until they achieved symptomatic remission.

**Table 1:** Demographic characteristics of SCZ and BPD samples.

	SCZ	BPD
*N*	49	49
Age (years)	30.4 ± 10.1	31.9 ± 9.4
Gender (male) *N* (%)	35 (71.4%)	21(42.9%)
BMI (kg/m^2^)	22.2 ± 3.7	23.4 ± 3.8
Family history *N* (%)	4 (8.2%)	7 (14.3%)
Smoker *N* (%)	4 (8.2%)	3 (6.1%)
Disease duration (years)	3.45 ± 5.05	6.45 ± 6.36
Marriage *N* (%)		
Single	29 (59.2%)	18 (36.7%)
Married	15 (30.6%)	25 (51.0%)
Divorced (widowed)	5 (10.2%)	6 (12.2%)
Education *N* (%)		
Middle school or below	27 (55.1%)	17 (34.7%)
Senior school	10 (20.4%)	16 (32.7%)
Collage or above	12 (24.5%)	16 (32.7%)

Continuous variables were presented as mean ± SD and categorical variables were shown as *N* (%).

*SCZ* Schizophrenia, *BPD* Bipolar disorder, *BMI* Body mass index.

**Table 2:** Clinical characteristics of SCZ and BPD participants.

	SCZ		BPD			
	Acute episode	Remission	Mania	Euthymia	Depression	Euthymia
*N*	49	42	41	32	9	9
WBC count (10^9^/L)	7.70 ± 2.18 (3.07–14.29)	7.06 ± 2.34 (4.16–14.50)^a^	7.94 ± 2.59 (3.76–14.66)	6.89 ± 2.01 (3.51–11.68)^b^	8.49 ± 1.63 (5.61–11.33)	6.67 ± 1.81 (3.83–9.58)^b^
Scale score	81.1 ± 11.4 (50–106)	43.6 ± 7.9 (30–62)	38.1 ± 7.6 (19–54)	16.4 ± 2.4 (6–20)	36.1 ± 3.5 (32–42)	7.8 ± 3.1(4–15)

One BPD patient was captured for mania, depression and euthymia and was arranged to both mania-euthymia group and depression-euthymia group.

Data were presented as mean ± SD (range). Scale rating scores were presented as PANSS, BRMS, HAMD for SCZ, mania and depression symptom evaluation, respectively.

*SCZ* Schizophrenia, *BPD* Bipolar disorder, *WBC* White blood cell, *PANSS* Positive and negative syndrome scale, *BRMS* Bech–rafaelsen mania rating scale, *HAMD* Hamilton depression rating scale.

^a^data missing for 5 patients; ^b^data missing for 1 patient.

This study was approved by the Ethical Committee of Hunan Provincial Brain Hospital (No. K2018014 and K2018037). Written informed consent was obtained from the patients or responsible family members of all participants after a full description of the study. All procedures complied with the Declaration of Helsinki.

### Clinical assessment

The symptom severity of patients was evaluated by rating scales. SCZ patients were assessed using Positive and Negative Syndrome Scale (PANSS) [20]. Bech-Rafaelsen Mania Rating Scale (BRMS) [21] and Hamilton Depression Rating Scale (HAMD) [22] were used to assess manic or depressive symptoms in BPD, respectively. A routine blood test was also conducted in all patients to obtain the white blood cell (WBC) count.

### Quantify immune-related factors

Fasting blood samples at two state time points (before and after treatment) were collected from patients in the morning to avoid circadian variation. The blood samples were drawn into K2 EDTA vacutainer tubes and immediately stored at 4 °C. The plasma was obtained by centrifugation at 1000 x *g* and 4 °C for 15 min and stored frozen at −80 °C until use.

A panel of 41 IRFs were assayed. The 41 factors were selected to maximize the number of factors that were available and could be assayed together in one customized Luminex panel. The panel contained subtypes of cytokines and cytokine receptors (interleukins, chemokines, interferons, tumor necrosis factors, growth factors) except for CRP (Table 3).

**Table 3:** Characteristics of 41 immune-related factors.

Category	IRF name	Number
Growth factor	β-NGF, FGF, VEGF-D	3
Interleukin	IL-1α, IL-1β, IL-18, IL-2, IL-15, IL-21, 1L-4, IL-5, IL-6, IL-31, IL-10, IL-12p70, IL-23, IL-13, IL-17A	15
Chemokine	CCL2, CCL3, CCL4, CCL11, CCL20, CCL24, CXCL1, CXCL2, CXCL9, CXCL8, CXCL10, SDF-1(CXCL12)	12
Interferon	IFN-β, IFN-γ, IFN-γR1	3
Tumor necrosis factor	TNF-α, TNF-R1, CD30, LT-α, BAFF	5
Others	GM-CSF, TSLP, CRP	3

*IRFs* Immune-related factors, *β-NGF* β-nerve growth factor, *VEGF-D* Vascular endothelial grown factor (VEGF)-D, *FGF* Fibroblast growth factor, *GM-CSF* Granulocyte-macrophage colony-stimulating factor, *TSLP* Thymic stromal lymphopoietin, *CRP* C-reactive protein.

These factors were measured simultaneously using the Human Magnetic Luminex Assay (R&D Systems, Minneapolis, MN, USA) according to the manufacturer’s instructions. Plasma samples were diluted in 1:2 (v/v) and read on a Luminex 200^TM^ System. Concentrations of our samples were calculated using a 5-parametric logistic (5-PL) calibration curve through Bio-Plex Manager software. All samples were randomly placed into the 96-well plates to avoid potential positional effects. Standards were run in duplicate. Four samples were assayed three times in different plates for evaluating inter-assay variation. Eight samples were loaded in duplicate on the same plate for evaluating intra-assay variation. The other samples were measured once.

To validate the results of the Luminex assay, CD30 and BAFF were re-measured using enzyme linked immunosorbent assay (ELISA). The human ELISA kits for BAFF (R&D Systems, Minneapolis, MN, USA), and CD30 (Novus Biologicals, Littleton, CO, USA) were used according to the manufacturer’s instructions. All standards and samples were assayed in duplicate. The absorbance was determined using a microplate reader (Bio-Rad, iMark) set at 450 nm.

### Quality control

Of the 41 IRFs assayed, two kinds of values were not precise quantification: missing values and the beyond standard range (BSR) values. The missing values were extremely high or low that were beyond the detection ability of the assay and were reported as “OOR>” or “OOR<”. The BSR values exceeded the upper or lower limits of standard samples but still yielded numerical values according to the standard curve. Details of the missing rate and BSR rate can be found in Supplementary Table 1. We excluded the IRFs with a sum of missing rate and BSR rate exceeding 60% (Supplementary Fig. 1). Finally, 11 factors (CCL20, CD30, IL-4, TNF-R1, BAFF, CCL2, CCL24, CXCL10, CXCL2, IL-18, and IFN-γR1) passed quality control in each of the three groups (SCZ group, BPD group, and psychosis group). Besides, IL-23 still survived in BPD group and psychosis group but not SCZ group. We assessed the accuracy of the panel for the 11 factors passing quality control; the average inter-assay CV (coefficient of variation) was 15.17% and the average intra-assay CV was 6.28%. For the missing values that were reported as “OOR<” or “OOR>”, a value of 0.5 x minimum value (1.25 x maximum value) replaced the extremely low (high) missing values.

### Statistical analysis

The IRFs passing quality control were log2 transformed for their quantifications. Potential confounders, including age, sex, body mass index (BMI), marriage, education, family history, smoking status, and batch effect, were controlled by the linear regression model. To compare the state differences of these IRF measures, we conducted two-tailed paired *t*-tests with samples two-state points (SCZ: *N* = 42; BPD: *N* = 41). Bonferroni method [23] was used to correct for multiple testing inflation. The corrected *p* < 0.05 was considered statistically significant. We used ELISA to validate the significantly altered IRFs. Pearson correlation was performed to determine the correlation between Luminex assay and ELISA. Significance for the two methods’ correlation was set at *p* < 0.05.

Pearson correlation was conducted to find the relationships among the altered IRFs which were continuous clinical variables with normal distribution. Spearman correlation was used to examine the correlation between IRFs in acute SCZ and BPD and other clinical features, including WBC count, symptom severity (psychiatric scale score), suicide behavior, suicide attempt, sleep quality, and disease duration, which were either categorical or quantitative variables not normally distributed. The relationship between changes of IRFs and symptom improvement (scale score change) and WBC count change were also investigated by Spearman correlation. Bonferroni correction was also used for the correlation analysis mentioned above. All analyses were performed using the R statistical software (version 3.6.2).

By G*Power 3.1, we calculated the statistical power for 11 factors that passed quality control given our sample size and predicted the sample size to achieve 80% power (*α* = 0.05/11, 0.0045) [24].

## Results

### State-related IRFs in SCZ or (and) BPD

**Fig. 1:**
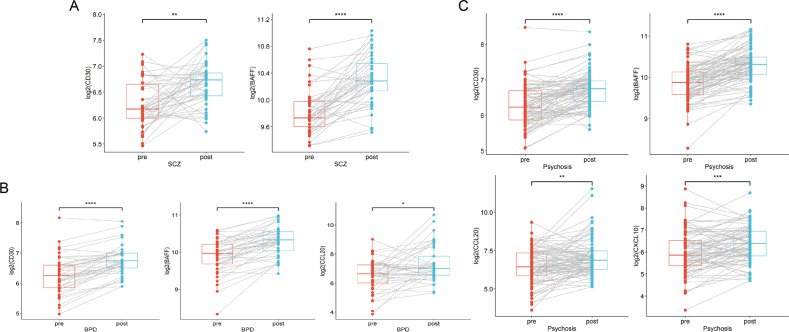
Significantly changed IRFs in SCZ or (and) BPD between acute episode and remission. **A** CD30 and BAFF expression were increased in SCZ patients after treatment (*N* = 42 pairs); **B** CD30, BAFF, and CCL20 expression were increased in BPD patients after treatment (*N* = 41 pairs); **C** CD30, BAFF, CCL20, and CXCL10 expression were increased in the total psychosis (SCZ+BPD) patients after treatment (*N* = 83 pairs). Paired *t* tests were completed to test for statistical significance and Bonferroni correction was performed for multiple comparisons in different groups. (*p < 0.05; **p < 0.01; ***p < 0.001; ****p < 0.0001). SCZ schizophrenia; BPD bipolar disorder.

After adjusting the covariates, the paired *t*-test in the SCZ group comparing remission to acute state identified significant elevation of CD30 (corrected *p* < 0.01) and BAFF (corrected *p* = 8.98e-09, Fig. 1A).

In the BPD group, CD30 (corrected *p* = 2.58e-09), BAFF (corrected *p* = 1.31e-07) and CCL20 (corrected *p* < 0.05) were significantly elevated in remission compared to acute state (Fig. 1B). Subsequently, BPD patients were split into two subgroups according to their initial mood state (mania or depression) in the acute episode. The mania subgroup showed significantly elevated expression of CD30 (corrected *p* = 1.02e-06) and BAFF (corrected *p* = 4.08e-07) at remission (Supplementary Fig. 2A). The depression subgroup only had significantly increased CD30 (corrected *p* < 0.01) level at remission, while BAFF had the trend of elevation at remission but with no statistical significance (nominal *p* < 0.05, corrected *p* > 0.05, Supplementary Fig. 2B).

The cross-disorder analysis combining SCZ and BPD showed significantly higher levels of CD30 (corrected *p* = 1.19e-10), BAFF (corrected *p* = 6.31e-16), CCL20 (corrected *p* < 0.01), and CXCL10 (corrected *p* < 0.001) at remission when compared to acute episode (Fig. 1C). All the significant findings survived Bonferroni correction.

### Correlation of the state-related IRFs

**Fig. 2:**
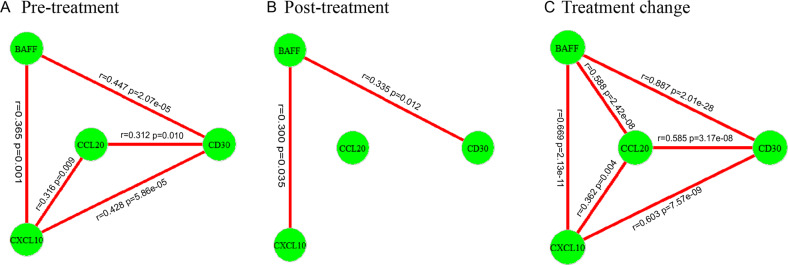
The correlation of state-related IRFs during treatment. **A** Pre-treatment correlation; **B** Post-treatment correlation; **C** Changes of treatment correlation. The altered IRF was considered to be a node in the network, and these nodes were connected by lines if there was evidence that they were associated by Pearson correlation analysis after Bonferroni correction. Pearson’s *r* and corrected *p* values were shown on the graph.

To investigate the correlation among the altered IRFs, we analyzed data of pre-treatment, post-treatment and pre-post difference separately for four differently expressed IRFs, CD30, BAFF, CXCL10, and CCL20. There were correlations among the IRFs in the pre-treatment and the post-treatment states, and among the changes of the four IRFs (Fig. 2).

### Correlation between state-related IRFs and clinical measures

We further tested correlation between the altered IRFs at the baseline (acute state) and their changes with the acute-state measures and changes of clinical measures, separately. For the IRF levels in acute episode, no factor correlated with clinical traits after multiple testing correction (corrected *p* > 0.05 for all comparisons) in the SCZ group. In the BPD group, significant positive correlation was identified between CD30 and sleep quality (Spearman’s *r*_*s*_ = 0.365, corrected *p* < 0.05, Supplementary Fig. 3A).

We further correlated the changes of IRFs with the changes of scale scores and WBC counts as these two factors were measured both pre- and post-treatment. In the SCZ group, the changes of CD30 (Spearman’s *r*_*s*_ = −0.395, nominal *p* < 0.05, Supplementary Fig. 3B) and BAFF (Spearman’s *r*_*s*_ = −0.366, nominal *p* < 0.05, Supplementary Fig. 3C) both showed negative correlation with the changes of WBC counts. However, the correlation could not survive multiple testing correction. In the BPD group, there was no significant correlation between the IRF alterations and the changes of scale scores or WBC counts (all nominal *p* > 0.05).

### Validation of CD30 and BAFF with ELISA

The most consistent findings of state-related IRFs BAFF and CD30 were re-measured by ELISA. The protein concentrations measured by ELISA and Luminex assays showed high correlation (CD30: Pearson’s *r* = 0.904, *p* < 2.2e-16; BAFF: Pearson’s *r* = 0.732, *p* < 2.2e-16; Supplementary Fig. 4).

### Power analysis

Since sufficiently powered studies are necessary for reliable results, we first calculated the power of the 11 IRFs passing quality control (Supplementary Table 2). In the SCZ group, CD30, BAFF, and CCL20 were well powered (>80%). In the BPD group, the same three IRFs plus CXCL10 were sufficiently powered (>80%). In all the psychosis patients combined, CD30, BAFF, CCL20, CXCL10, and IFN-γ R1 achieved enough power (>80%). We may need to increase sample size to improve the power to study the other underpowered IRFs according to Supplementary Table 2.

## Discussion

In this study, we investigated the peripheral IRF alterations between acute and remission states in SCZ and BPD patients during regular in-patient medication treatment. After treatment, SCZ patients showed increased CD30 and BAFF levels while BPD patients showed elevated CD30, BAFF, and CCL20 expression. When combining the two disorder groups for cross-disorder analysis, four cytokines or cytokine receptors CD30, BAFF, CXCL10, and CCL20, showed significant alterations after treatment.

### CD30, BAFF, CCL20, and CXCL10 were state-related IRFs for SCZ and BPD

Of the 11 IRFs passing quality control, four factors CD30, BAFF, CXCL10, and CCL20 showed stated-related alterations in SCZ and BPD that survived strict Bonferroni correction. The statistical power of these altered IRFs in the three groups (SCZ, BPD, all psychosis) was above 80%. CD30 and BAFF were both altered in SCZ and BPD, which implies the two factors might reflect common immune states related to the treatment of the two disorders. In contrast, CCL20 was increased only in BPD patients after treatment. This indicated that CCL20 might be specific to BPD and could possibly be distinguished from SCZ. CXCL10 change was not significant in either SCZ and BPD alone, which might be related to its relatively small effect size.

CD30 and BAFF belong to the TNF superfamily, which not only triggers apoptosis but also affects immune response partially by activating NF-κB signaling pathways [25]. Yousri El Kissi et al. reported decreased BAFF serum level in drug-free acute schizophrenia compared to healthy control [26]. However, there is no previous study of CD30 and BAFF changes in plasma of treated SCZ and BPD patients. Our result is in line with the results of Yousri El Kissi et al., suggesting the treatment might rescue the reduction of BAFF in patients.

The chemokine family consists of a large number of ligands and receptors that can participate in the development of the immune system and inflammatory responses [27]. Previous studies have shown that chemokines are involved in psychiatric disorders [28]. In our study, we identified two chemokines, CXCL10 and CCL20, as state-related factors in treatment of SCZ and BPD. CXCL10 acts through its receptor CXCR3 and functions as a major chemoattractant for activated T cells and natural killer cells [29]. CCL20 interacts with its chemokine receptor CCR6 and the CCL20-CCR6 axis can regulate T-B cell immunobiology [30]. The psychiatric disorder increase the risk of suicide behavior [31]. A previous study showed that CCL20 of the dorsolateral prefrontal cortex were significantly decreased in suicide completers (some of them accompanied with comorbid psychiatric disorder) compared to control (individuals died for other reasons besides suicide) [32]. Our study was consistent with this and implied that the treatment might rescue the reduction of CCL20 in patients.

### Correlation among the IRFs

The 41 IRFs are messenger molecules and can promote cell communication in the immune system [33]. They cooperate with each other and form complex networks.

IRFs of the same family can influence each other. For example, TNF members can drive either co-stimulation or co-inhibition of the immune response [25]. We found that BAFF and CD30 showed a strong correlation in SCZ and BPD patients at all measures, including pre- and post-treatment measures and changes in between, showing their coordinated responses. IRFs derived from the TNF family also show synergistic interaction with chemokine family members and thus enhance the inflammatory response [34]. Our findings that two TNF members (BAFF and CD30) were positively correlated with chemokine members (CXCL10 and CCL20) at acute and remission states and also for the changes between the two states are consistent with the findings of Gouwy et al. [34]. In total, these factors were highly coordinated in the treatment response.

### Treatment may influence the immune level

In the current study, patients with SCZ or BPD received various types of monotherapy with antipsychotics, mood stabilizers, or antidepressants according to their clinical situation. Despite the medication differences, patients exhibited surprisingly similar patterns of immune factor changes. However, we cannot conclude a causal effect between the IRF alterations and the clinical improvement after treatment. In the limited clinical phenotypes we studied, we did not detect direct correlation between IRF changes and improvement of clinical measures. IRF changes may be associated with other clinical variables that we did not evaluate, or the effect size of IRF on clinical change may be too small to be detected in our relatively small samples. It is still possible that these IRFs partially contribute to the clinical improvement, but larger samples would be needed to detect the small effects. More clinical measures could be investigated to solve the puzzles regarding how these IRF changes are related to the treatment effects.

### Limitations

There are several limitations that should be considered in the interpretation of our results. First, SCZ and BPD patients received different classes and doses of drugs, which made it difficult to study changes associated with specific medication. Interestingly, we detected changes across disorders regardless of the diverse treatments, indicating the robustness of our findings. Second, some previously reported state-related factors, such as TGF-β were not included in our panel due to the incompatibility in the Luminex assay design [16]. Moreover, of the 41 factors selected, 30 were filtered out due to high missing rate or BSR rate, like CRP, IL-4, IL-6, which were likely state-related IRFs according to our review of previous publications [18]. Therefore, we cannot ignore those 30 IRFs that were filtered out of this study and they deserve further investigation. Lastly, we did not recruit a control group in parallel comparison so the findings only pertain to changes in the patients. We are assuming that these cytokines are relatively stable in healthy people, which is very likely true, particularly for BAFF as reported before [35–37]. There is no reason to expect them to consistently change in healthy controls within one month without medication intervention. This certainly should be validated by experiments.

## Conclusion

In summary, employing the Luminex platform, we have identified cytokine-related alterations in SCZ and BPD between acute state and remission in Chinese patients. Four cytokines or cytokine receptors showed significant changes after treatment. CD30 and BAFF are the universal biomarkers for the treatment response of SCZ and BPD while CCL20 may be specific for BPD treatment. CXCL10 is only significant in the combined cross-disorder data, but not in subgroups. These four cytokines or cytokine receptors may be good candidates for developing biomarkers for monitoring treatment response, and for studying the involvement of peripheral immune systems in psychosis.

## Supplementary information

Supplementary figure legends and tables

Supplementary figure 1

Supplementary figure 2

Supplementary figure 3

Supplementary figure 4
