# Supplementary material for: Changes of immune-related factors in the blood of schizophrenia and bipolar disorder patients receiving monotherapy
Source: Transl Psychiatry. 2022 May 26;12:212. doi: 10.1038/s41398-022-01968-0 (PMC9135722; doi:10.1038/s41398-022-01968-0)
Supplement: Supplementary file 1 — Supplementary figure legends and tables [file 41398_2022_1968_MOESM1_ESM.docx]

**Supplementary Figure Legends**

**Supplementary Figure 1. IRFs passed quality control with different threshold**

Threshold are the sum of missing rate and beyond standard range (BSR) rate of IRFs. IRFs passed quality control mean the sum of missing rate and BSR rate of IRFs are less than or equal to the threshold.

**Supplementary Figure 2. Significantly changed IRFs in BPD subgroup between acute episode and remission**

(A) CD30 and BAFF expression were increased in mania patients after treatment (N = 32 pairs); (B) CD30 expression was increased after treatment in depression patients but not BAFF although they were in the same trend (N = 9 pairs). Paired t test was carried out to check for statistical significance and Bonferroni correction was performed for multiple comparisons in different groups. (*corrected *p* < 0.05; **corrected *p* < 0.01; ***corrected *p* < 0.001; ****corrected *p* < 0.0001; ns, not significant).

**Supplementary Figure 3. Correlation between the state-related IRFs and clinical traits**

(A) CD30 correlated with sleep quality in BPD; (B) Changes of CD30 correlated with changes of WBC count in SCZ; (C) Changes of BAFF correlated with changes of WBC count in SCZ. WBC, whole blood cell. Nominal *p*, Spearman correlation without Bonferroni correction; corrected *p,* Spearman correlation after Bonferroni correction.

**Supplementary Figure 4. ELISA validation of BAFF and CD30**

Significant correlations were observed in CD30 (A: Pearson’s *r* = 0.732, *p* < 2.2e-16) and BAFF (B: Pearson’s *r* = 0.904, p < 2.2e-16) concentrations between Luminex assay and ELISA. ELISA, enzyme linked immunosorbent assay.

**Supplementary Tables**

**Supplementary Table 1. Quality evaluation of 41 immune-related factors**

|  | SCZ missing  rate (%) | SCZ  BSR  rate (%) | BPD missing rate (%) | BPD  BSR  rate (%) | Psychosis missing rate (%) | Psychosis BSR  rate (%) |
| --- | --- | --- | --- | --- | --- | --- |
| CCL11 | 21.98 | 54.95 | 15.56 | 60 | 18.78 | 57.46 |
| CCL20 | 0 | 0 | 1.11 | 2.22 | 0.55 | 1.1 |
| CCL3 | 45.05 | 53.85 | 28.89 | 70 | 37.02 | 61.88 |
| CD30 | 0 | 0 | 0 | 0 | 0 | 0 |
| CXCL1 | 49.45 | 16.48 | 48.89 | 20 | 49.17 | 18.23 |
| SDF-1 | 94.51 | 0 | 91.11 | 4.44 | 92.82 | 2.21 |
| CXCL8 | 50.55 | 36.26 | 44.44 | 44.44 | 47.51 | 40.33 |
| FGF | 59.34 | 21.98 | 53.33 | 22.22 | 56.35 | 22.1 |
| GM-CSF | 78.02 | 21.98 | 53.33 | 46.67 | 65.75 | 34.25 |
| IFN-γ | 59.34 | 40.66 | 35.56 | 62.22 | 47.51 | 51.38 |
| IL-1α | 39.56 | 47.25 | 20 | 52.22 | 29.83 | 49.72 |
| IL-10 | 91.21 | 4.4 | 88.89 | 8.89 | 90.06 | 6.63 |
| IL-13 | 39.56 | 56.04 | 38.89 | 56.67 | 39.23 | 56.35 |
| IL-17A | 53.85 | 43.96 | 63.33 | 36.67 | 58.56 | 40.33 |
| IL-23 | 27.47 | 37.36 | 10 | 37.78 | 18.78 | 37.57 |
| IL-4 | 15.38 | 17.58 | 16.67 | 17.78 | 16.02 | 17.68 |
| IL-6 | 10.99 | 84.62 | 6.67 | 87.78 | 8.84 | 86.19 |
| TNF-RI | 0 | 0 | 0 | 0 | 0 | 0 |
| TSLP | 52.75 | 47.25 | 30 | 70 | 41.44 | 58.56 |
| BAFF | 0 | 0 | 0 | 0 | 0 | 0 |
| β-NGF | 6.59 | 93.41 | 0 | 96.67 | 3.31 | 95.03 |
| CCL2 | 0 | 2.2 | 0 | 0 | 0 | 1.1 |
| CCL24 | 0 | 0 | 0 | 0 | 0 | 0 |
| CCL4 | 1.1 | 60.44 | 0 | 64.44 | 0.55 | 62.43 |
| CRP | 0 | 97.8 | 0 | 96.67 | 0 | 97.24 |
| CXCL10 | 0 | 0 | 0 | 0 | 0 | 0 |
| CXCL2 | 17.58 | 24.18 | 22.22 | 25.56 | 19.89 | 24.86 |
| CXCL9 | 52.75 | 46.15 | 31.11 | 68.89 | 41.99 | 57.46 |
| IL-18 | 0 | 1.1 | 0 | 3.33 | 0 | 2.21 |
| IFN-β | 67.03 | 32.97 | 44.44 | 55.56 | 55.8 | 44.2 |
| IFN-γ R1 | 0 | 0 | 0 | 0 | 0 | 0 |
| IL-1β | 48.35 | 46.15 | 32.22 | 65.56 | 40.33 | 55.8 |
| IL-12p70 | 50.55 | 49.45 | 30 | 70 | 40.33 | 59.67 |
| IL-15 | 70.33 | 29.67 | 46.67 | 51.11 | 58.56 | 40.33 |
| IL-21 | 0 | 98.9 | 0 | 100 | 0 | 99.45 |
| IL-31 | 91.21 | 8.79 | 86.67 | 13.33 | 88.95 | 11.05 |
| IL-5 | 38.46 | 61.54 | 25.56 | 74.44 | 32.04 | 67.96 |
| LT-α | 81.32 | 16.48 | 85.56 | 13.33 | 83.43 | 14.92 |
| TNF-α | 28.57 | 71.43 | 20 | 80 | 24.31 | 75.69 |
| VEGF-D | 35.16 | 61.54 | 16.67 | 83.33 | 25.97 | 72.38 |
| IL-2 | 64.84 | 3.3 | 61.11 | 7.78 | 62.98 | 5.52 |

Note: BSR, beyond standard range

**Supplementary Table 2. Power analysis**

|  |  | CCL20 | CD30 | IL-4 | TNF-RI | BAFF | CCL2 | CCL24 | CXCL10 | CXCL2 | IL-18 | IFN-γ R1 |
| --- | --- | --- | --- | --- | --- | --- | --- | --- | --- | --- | --- | --- |
| SCZ | effect size | 0.27 | 0.64 | 0.27 | 0.03 | 1.22 | 0.10 | 0.02 | 0.45 | 0.16 | 0.11 | 0.31 |
|  | power (%) | 34 | 100 | 32 | 1 | 100 | 3 | 1 | 88 | 8 | 3 | 47 |
|  | sample size | 189 | 38 | 196 | 17186 | 14 | 1304 | 34075 | 71 | 546 | 1202 | 145 |
| BPD | effect size | 0.53 | 1.32 | 0.11 | 0.15 | 1.12 | 0.29 | 0.33 | 0.43 | 0.08 | 0.16 | 0.29 |
|  | power (%) | 96 | 100 | 3 | 6 | 100 | 38 | 54 | 82 | 2 | 8 | 39 |
|  | sample size | 54 | 12 | 1036 | 649 | 15 | 167 | 126 | 79 | 1905 | 524 | 167 |
| Psychosis | effect size | 0.41 | 0.87 | 0.18 | 0.08 | 1.16 | 0.11 | 0.14 | 0.47 | 0.07 | 0.12 | 0.30 |
|  | power (%) | 99 | 100 | 29 | 4 | 100 | 7 | 15 | 100 | 3 | 10 | 84 |
|  | sample size | 84 | 23 | 422 | 1992 | 15 | 1156 | 691 | 66 | 2766 | 887 | 154 |

Note: α=0.0045; Sample size was predicted according to 80% power in each group.
