# Supplementary figures and images for: Changes of immune-related factors in the blood of schizophrenia and bipolar disorder patients receiving monotherapy
Source: Transl Psychiatry. 2022 May 26;12:212. doi: 10.1038/s41398-022-01968-0 (PMC9135722; doi:10.1038/s41398-022-01968-0)

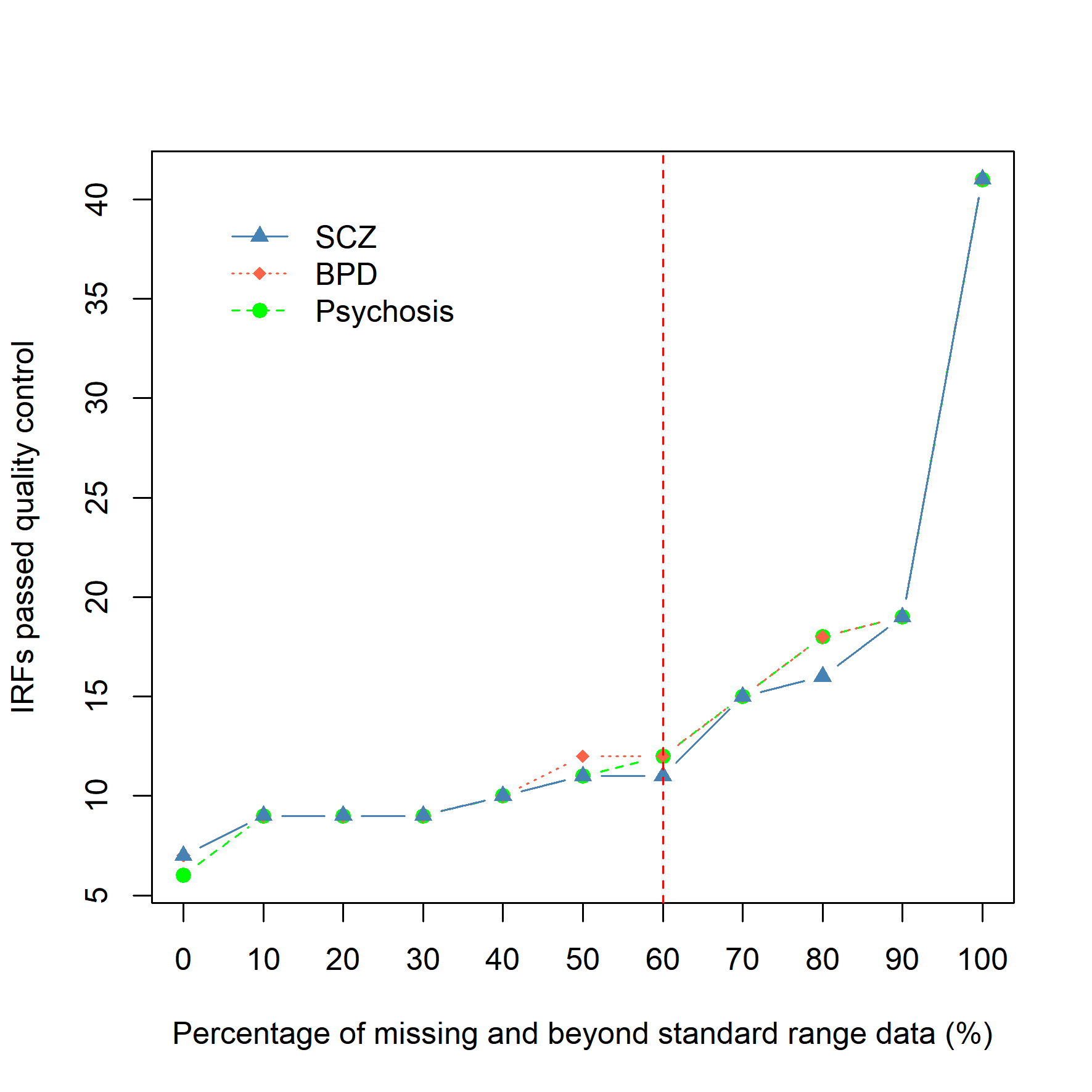

Supplement: Supplementary file 2 — Supplementary figure 1 [file 41398_2022_1968_MOESM2_ESM.tif]

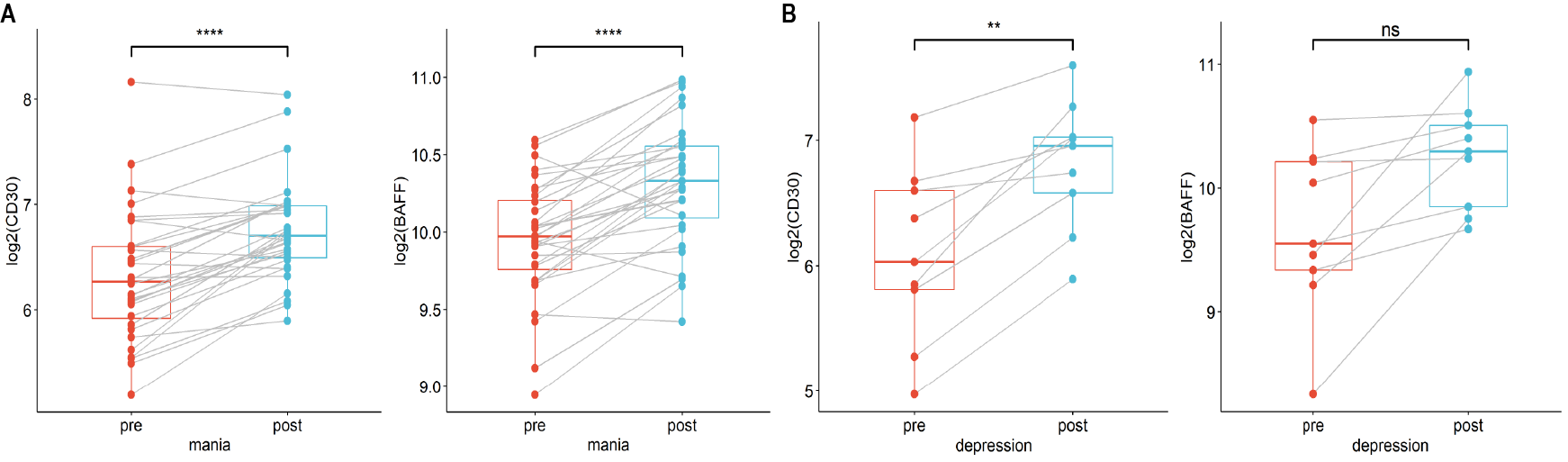

Supplement: Supplementary file 3 — Supplementary figure 2 [file 41398_2022_1968_MOESM3_ESM.tif]

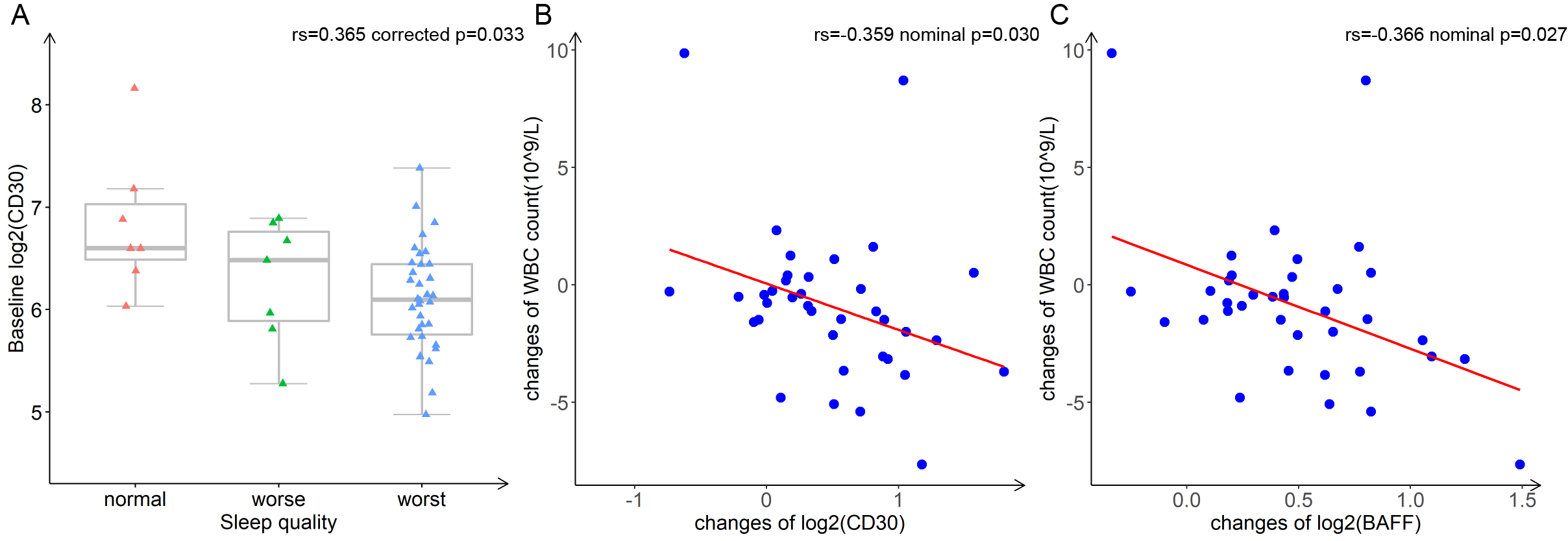

Supplement: Supplementary file 4 — Supplementary figure 3 [file 41398_2022_1968_MOESM4_ESM.tif]

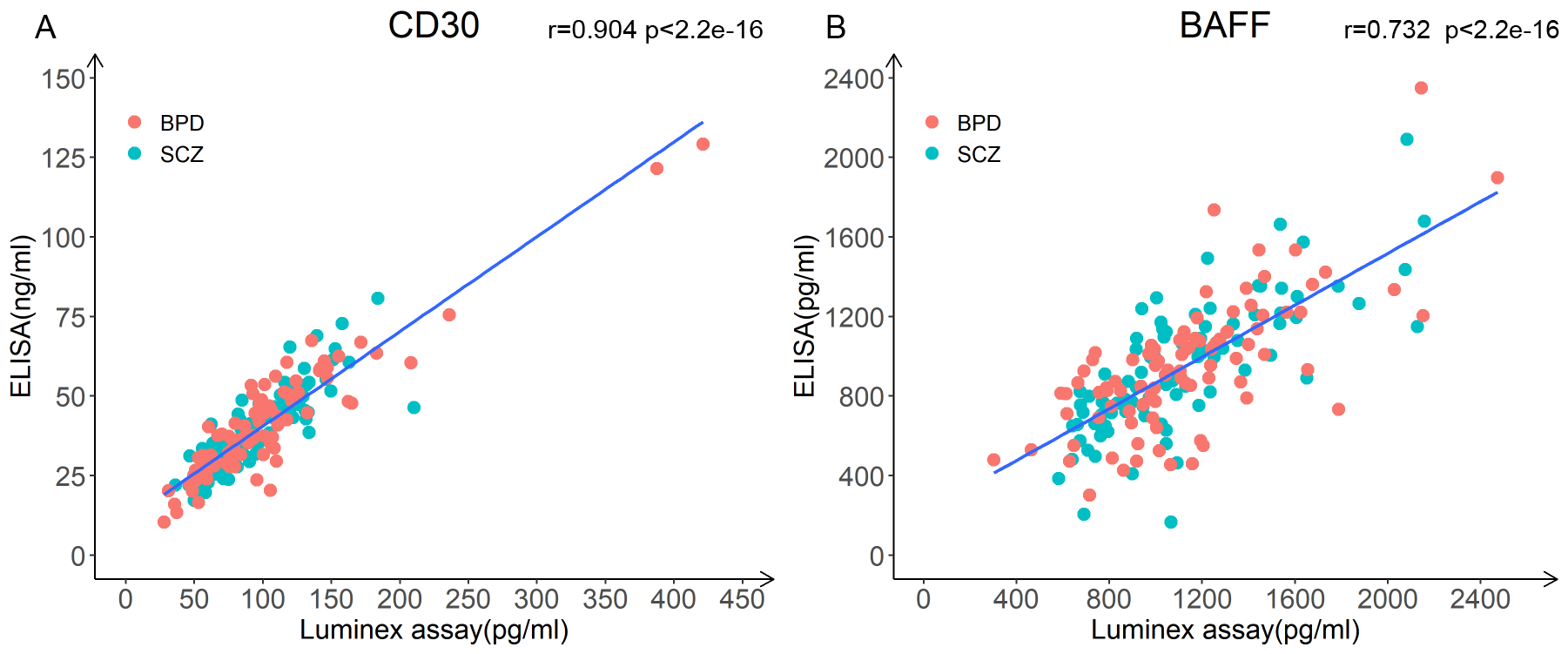

Supplement: Supplementary file 5 — Supplementary figure 4 [file 41398_2022_1968_MOESM5_ESM.tif]
